# Supplementary material for: In Vitro Hepatic Assessment of Cineole and Its Derivatives in Common Brushtail Possums (Trichosurus vulpecula) and Rodents
Source: Biology (Basel). 2021 Dec 15;10(12):1326. doi: 10.3390/biology10121326 (PMC8698377; doi:10.3390/biology10121326)
Supplement: Supplementary file 1 [file biology-10-01326-s001.zip › Figure S1.pdf]

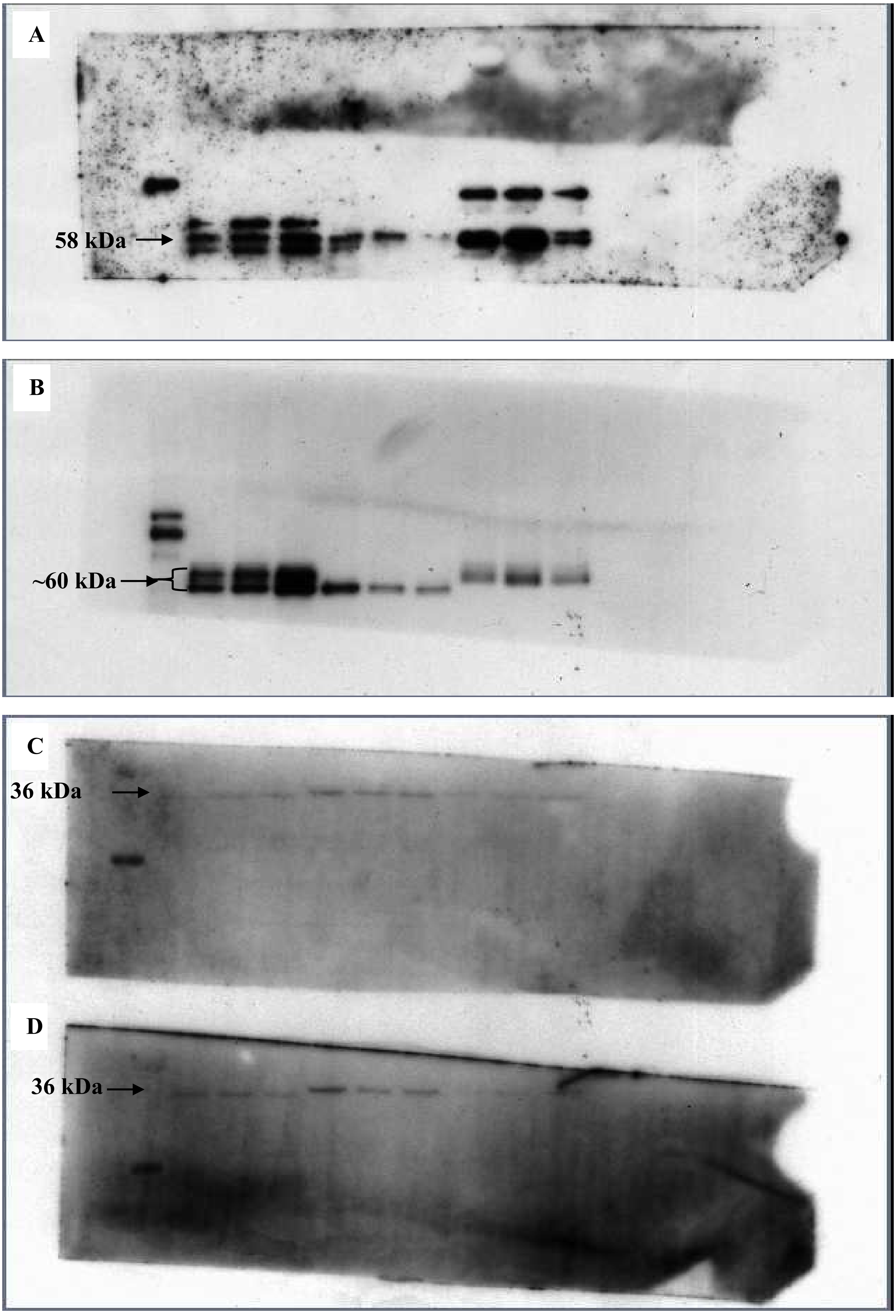

Figure S1. CYP3A and UGT2B4 polypeptide levels in mouse, rat and possum hepatic microsomes (n = 3). (**A**,**B**)—GAPDH was used as a control for CYP3A and UGT2B4 (**C**,**D**). Western blots were visualised using a CL-XPosure Film (ThermoFisher, Albany, New Zealand). The X-ray film was digitalised using a BioRad densitometer GS710.
